# Untreated depression and anxiety in patients with common skin diseases: a cross-sectional study in China

**DOI:** 10.3389/fpsyg.2023.1150998

**Published:** 2023-05-16

**Authors:** Tao-Ran Tang, Mi Wang, Hong Li, Song-Chun Yang, Cheng-Cheng Zhang, Wen-Rui Lin, Xin-Chen Ke, Han-Yi Zhang, Juan Su, Shi-Lin Zhu

**Affiliations:** ^1^School of Nursing, Hunan University of Chinese Medicine, Hunan, Changsha, China; ^2^Department of Nursing, The Second Affiliated Hospital of Hunan University of Chinese Medicine, Hunan, Changsha, China; ^3^Department of Dermatology, Xiangya Hospital, Central South University, Hunan, Changsha, China; ^4^Department of Social Medicine and Health Management, School of Public Health, Central South University, Hunan, Changsha, China

**Keywords:** depression, anxiety, skin disease, mental health service, web-based, survey

## Abstract

**Objective:**

The study aimed to analyze the current status and reasons for the neglect of the psychological need of patients with common skin diseases.

**Methods:**

This cross-sectional study was conducted in China using an online self-assessment questionnaire distributed via social media. Demographic, clinical and psychological data were collected, and the main outcomes, i.e., depression (evaluated using the 9-item Patient Health Questionnaire, PHQ-9) and anxiety (evaluated using Generalized Anxiety Disorder-7, GAD-7). Multivariate regression analysis was used for the prediction of variates of mental health service seeking behaviors.

**Results:**

A total of 1,010 patients participated in the survey, and 273 (27.0%) patients met the “with need” criteria, i.e., having the need for mental health intervention but not being treated. In the multivariate regression model, income (OR = 0.80, 95%CI: 0.65–0.99), duration of disease (OR = 0.68, 95%CI: 0.49–0.95) and suicide ideation (OR = 2.10, 95%CI: 1.14–3.87) were significant factors. For patients who did not receive mental health care, the lack of knowledge about the availability of mental health services, lack of knowledge of where to seek help, concerns about the side effects of treatment, failure to seek treatment for severe skin diseases, and absence of current psychological distress were associated with their need for psychological intervention.

**Conclusion:**

This study examined the current status of the need for psychological intervention and the reasons why the need was unmet in patients with skin diseases. Due to the confusion and a lack of knowledge about their mental health issues, mental health services are often underutilized.

## 1. Introduction

The China Mental Health Survey (CMHS) from 2012 to 2015 showed that the prevalence of depression was 6.8% and the prevalence of anxiety was 7.6% in Chinese adults (Huang et al., 2019; Lu et al., 2021). At the level of primary medical care, patients are less likely to seek psychological intervention due to lack of knowledge, misunderstanding, concerns about leakage of private information, and unavailability of professional services (Zhou and Xiao, 2022). Meanwhile, due to the confusion between mental and physical problems and worries about stigma, the history of treatment for mental disorders might be underestimated during surveys in China (Huang et al., 2016). These factors have contributed to the difficulty in investigating the incidence of mental disorders and treatment status of patients. National Essential Public Health Services Programs showed that the rate of standardized management of patients with severe mental disorders was 89.2% in 2019, and there are still cases of non-standard or poor management (Xiao et al., 2017; Lili, 2022). Thus, the popularization of mental health services in China is faced with a variety of hurdles such as lack of knowledge of patients, stigmatization, and lack of quality assurance for such services.

The prevalence of skin diseases in China reached 26.0% in 2019, making skin diseases the 7th leading cause contributing to increased years lived with disability globally (Peng et al., 2021). Skin diseases can seriously affect the patients’ quality of life and lead to negative emotions (Balieva et al., 2017). A survey in Europe found that the incidence of depression and anxiety reached 10.1 and 17.2%, respectively, among patients with skin diseases (Dalgard et al., 2015). According to a survey in the UK, psychological intervention and a combination of psychological and dermatological treatments are in great demand among patients with skin diseases due to decreased quality of life and lack of social recognition (Edward, 2020). A study in China found that the rate of suicidal ideation and behaviors is high among patients with psoriasis, but the proportion of psychologically untreated patients reached 88.9% (Chen et al., 2019), indicating that a large number of Chinese patients with physical and mental problems are not receiving timely treatment. At present, studies on patients with skin diseases are focusing on the analysis of the status quo of impaired quality of life and adverse psychological events, relevant interventions, as well as challenges for patients to seek mental health care, such as concerns for adverse drug reactions and underrating the importance of mental health (Achtman et al., 2016; Arévalo-Bermúdez et al., 2018; Iani et al., 2020; Domogalla et al., 2021). Meanwhile, there are few analyses on factors leading to patients’ non-treatment-seeking in psychology and the underlying reasons, and there is also a lack of investigations on the persistent underutilization of mental health services by patients with skin diseases as well as the increasing need for psychological and dermatological interventions. At present, although psycho-dermatology clinics are implementing interdisciplinary collaboration and primary prevention, to some extent, the user population needs to be expanded (Edward, 2020; Patel and Jafferany, 2020). Therefore, it is urgently needed to increase the attention to the mental health of patients with skin diseases and promote the patients’ utilization of mental health care.

The present study examined the depression and anxiety of patients with skin diseases who needed psychological intervention using the 9-item Patient Health Questionnaire (PHQ-9) and Generalized Anxiety Disorder-7 (GAD-7). This study also included participants with less severe symptoms (i.e., with low scores of the PHQ-9 and GAD-7) but on psychiatric medications and/or talk therapy (treatment provided by a counselor in a specialist setting) (Achtman et al., 2016). We aimed to investigate the need for intervention of depression and anxiety in Chinese patients with skin diseases and analyze the reason for their non-treatment-seeking, in order to improve the patients’ understanding of the relationship between skin diseases and psychological distress, improve the patients’ quality of life and disease outcomes, and promote the training of multidisciplinary professionals and integration of institutions.

## 2. Materials and methods

### 2.1. Participants

An online survey was created and distributed via social media platforms (WeChat groups and teledermatology platforms). WeChat groups consisted of patients who were diagnosed as having skin disease in the Department of Dermatology, Xiangya Hospital, Changsha, China. Teledermatology platform presented an application where patients, who had visited before in the department of dermatology, could communicate with the dermatologist online. All patients using WeChat could see this survey, and answer the questionnaire by scanning the Quick Response code (QR code) of the questionnaire address or clicking the relevant link.

Individuals diagnosed with any skin disease aged ≥18 years were potential eligible participants, and those with comorbidities, such as malignancies, impaired cardiac, respiratory or renal function, or severe psychiatric conditions that greatly affected the adherence to the study, were excluded. All diagnose were confirmed from self-reported visits with the previous dermatologist. Each IP address was allowed to submit answers only once to prevent repeated submissions. Completion of all questions was required before submission of the questionnaire. The participants could withdraw at any time they feel uncomfortable about any question. A total of 1,079 patients participated in the survey between April 14, 2022 and May 17, 2022, with 31 exiting before completion and 1,048 having completed the questionnaire. Among the participants who successfully submitted their answers, 21 with undefined diagnoses and 17 diagnosed with conditions other than skin diseases were excluded. This study was reviewed and approved by the ethics committee of Xiangya Hospital, Central South University (Changsha, China; approval number: 202206315); written electronic informed consents form were obtained from all participants before they start to do online survey.

### 2.2. Measures

#### 2.2.1. Demographic and clinical variables

The demographic and clinical variables included annual income (<US $1,500, US $1,500–7,499, US $7,500–14,999, US $15,000–29,999, ≥US $30,000), duration of disease and treatment delay (<1 year, 1–5 years, 6–10 years, >10 years), treatment satisfaction in dermatology (not at all satisfied, moderately satisfied, satisfied, very satisfied) and medical costs in dermatology (<US $1,500, US $1,500–7,499, US $7,500–14,999, US $15,000–29,999, ≥US $30,000). Adherence to dermatological treatment was defined by one item: “Have you been adherent to the dermatological treatment prescribed (not adherent, adherent, no treatment prescribed).” Any experience of not applying the dermatological therapy on time, with treatment prescribed, was regarded as not adherent.

#### 2.2.2. Psychological variables

Stigmatization was assessed using a 6-item scale inquiring about the level of perceived stigmatization due to skin diseases (Lu et al., 2003). 4-point Likert scale (not, a little, strongly, totally) was used. Significant stigmatization was indicated by a positive score (i.e., ≥1) on any least 1 of the 6 items. The Cronbach α coefficient was 0.88 in the validated study among patients with psoriasis (Łakuta et al., 2018), and 0.94 in the present study.

The impact of skin diseases on sleep and self-esteem were self-rated by patients on a 4-point Likert scale. Any perceived impact on sleep or self-esteem was regarded as significant. Suicide ideation and behavior were also self-rated by the patients on a 4-point Likert scale, using a single question: “Have you had suicide ideation/behavior due to skin disease.” Suicide behavior defined as a combination of suicide attempts and suicide ideation (Silverman et al., 2007). Any experience of suicide behavior or ideation was regarded as significant.

#### 2.2.3. Patient-reported outcomes

PHQ-9 and GAD-7 were used to access the patients’ depression and anxiety during the past 2 weeks (Tian et al., 2019). According to the guidelines for these scales, the cutoff scores of PHQ-9 and GAD-7 were set at 10 and 8, i.e., a PHQ-9 score of ≥10 and a GAD-7 score of ≥8 indicated that the patient needed intervention for depression and anxiety, respectively (Kroenke and Spitzer, 2002; Kroenke et al., 2007; Huang et al., 2020). Patients with a score lower than the cutoff score on these scales but on psychiatric medications and/or talk therapy were also regarded as needing psychological intervention (Achtman et al., 2016). The Cronbach α coefficients of PHQ-9 and GAD-7 in the simplified Chinese version were, respectively, 0.86 and 0.89 (Wang et al., 2014; Tong et al., 2016). The Cronbach α coefficients of PHQ-9 and GAD-7 were 0.91 and 0.95, respectively, in the present study.

The utilization of mental health services was described using a single question: “Have you ever received any mental health care due to psychological distress caused by skin disease?” Categories of mental health care referred to psychiatric medication, talk therapy and/or alternative treatment. Patients who received alternative therapy were not identified as adequately treated or needing psychological intervention (Achtman et al., 2016; Lu et al., 2021). The clinical assessment of the reasons for non-treatment-seeking in psychology was conducted by a registered psychiatrist using a semi-structured interview modeled after the SCID (First and Gibbon, 2004). The options for the reason in the questionnaire included lack of knowledge about the availability of and accessibility to mental health services, underestimation of the effect of mental health care on skin diseases, worries about the side effects of treatment, failure to seek help timely when the skin disease became severe, having been used to the negative impact of skin diseases, and absence of current psychological distress.

#### 2.2.4. Statistical analysis

The data were exported from the online survey system and analyzed using the IBM SPSS Statistics 26.0. Normally distributed continuous variables were presented as mean ± SD and categorical variables were presented as the number of cases (percentage). To compare the covariates of mental health care seeking by patients with need for psychological intervention, we used Chi-square test or Fisher’s exact test for dichotomous variables. The interaction effects of the exposure variables were examined. The associations between mental health care seeking and variables was evaluated using multivariable logistic regression adjusted for potential confounders. Sex and age were adjusted as covariates. The effect sizes of the associations were presented as adjusted odds ratios (aORs) and 95% CIs. For all the analyses, *p* < 0.05 indicated that the difference was statistically significant.

## 3. Results

### 3.1. Demographic and clinical characteristics

A total of 1,010 valid questionnaires were collected and analyzed. All of the participants were 18–80 years of age, with a mean age of 33.7 ± 12.3 years. Among them, 62.3% were female and 51.4% were married. 42.1% of the patients had a university degree, while 2.8% had a highest education level of primary school. The most reported type of skin diseases was skin appendage disorders (35.1%), followed by allergic skin diseases (31.7%), papulosquamous disorders (18.3%), hair disorders (11.8%), infectious skin diseases (10.5%), skin tumors (5.1%), neuropsychiatric skin diseases (4.6%), pigmentary disorders (4.0%), other skin diseases (1.3%), connective tissue diseases (1.0%), and herpes (0.6%). With regard to annual income, 27.4% of the patients had an income of US $ 7,500–14,999, and 10.8% had an income of < US $ 1,500, while in terms of medical cost, 57.0% of the patients paid <US $ 1,500 for their skin diseases and 1.9% paid a large sum of ≥US $ 30,000 to treat their skin diseases. Nearly half of the patients (41.2%) had an illness duration of 1–5 years and 13.5% had a duration of 6–10 years; however, 37.9% of the participants had a treatment delay of <1 year, and 12.8% had over 10 years of treatment delay (Table 1).

**Table 1:** Patient characteristics (*N* = 1,010).

Characteristic	Value
Age (years), mean ± SD	33.7 ± 12.3
Sex, n (%)	
Male	381 (37.7)
Female	629 (62.3)
Marital status, n (%)	
Unmarried	463 (45.8)
Married	519 (51.4)
Divorced	22 (2.2)
Widowed	6 (0.6)
Working status, n (%)	
Employed	575 (56.9)
Unemployed	121 (12.0)
Retired	52 (5.1)
Studying	262 (25.9)
Education, n (%)	
Primary school or below	28 (2.8)
Secondary school	80 (7.9)
High school	92 (9.1)
College	210(20.8)
University	425 (42.1)
Master or above	175 (17.4)
Skin disease, n (%)	
Skin appendage disorders	355 (35.1)
Allergic skin diseases	320 (31.7)
Papulosquamous disorders	185 (18.3)
Hair disorders	119 (11.8)
Infectious skin diseases	106 (10.5)
Skin tumors	52 (5.1)
Neuropsychiatric skin diseases	46 (4.6)
Pigmentary disorders	40 (4.0)
Connective tissue diseases	10 (1.0)
Herpes	6 (0.6)
Others	13 (1.3)
Annual income (US $)^a^, n (%)	
<1,500	109 (10.8)
1,500–7,499	241 (23.9)
7,500–14,999	277 (27.4)
15,000–29,999	222 (22.0)
≥30,000	161 (15.9)
Medical cost (US $)^a^, n (%)	
<10,000	576 (57.0)
10,000–49,999	223 (22.1)
50,000–99,999	111 (11.0)
100,000–199,999	81 (8.0)
≥200,000	19 (1.9)
Duration of disease (years), n (%)	
<1	295 (29.2)
1–5	416 (41.2)
6–10	136 (13.5)
>10	163 (16.1)
Treatment delay (years), n (%)	
<1	383 (37.9)
1–5	370 (36.6)
6–10	128 (12.7)
>10	129 (12.8)
Treatment adherence	
Non-adherent	166 (45.1)
Adherent/no treatment prescribed	202 (54.9)
Suicide ideation due to skin diseases, n (%)	237 (23.5)
PHQ-9, n (%)	
<10	758 (75.0)
≥10	252 (25.0)
GAD-7, n (%)	
<8	776 (76.8)
≥8	234 (23.2)
Mental health care seeking due to psychological distress caused by skin diseases, n (%)	
No	895 (88.6)
Yes	115 (11.4)

PHQ-9, Patient Health Questionnaire-9; GAD-7, Generalized Anxiety Disorder-7.

^a^US $0.15 = 1 Chinese Yuan (CNY).

**Figure 1:**
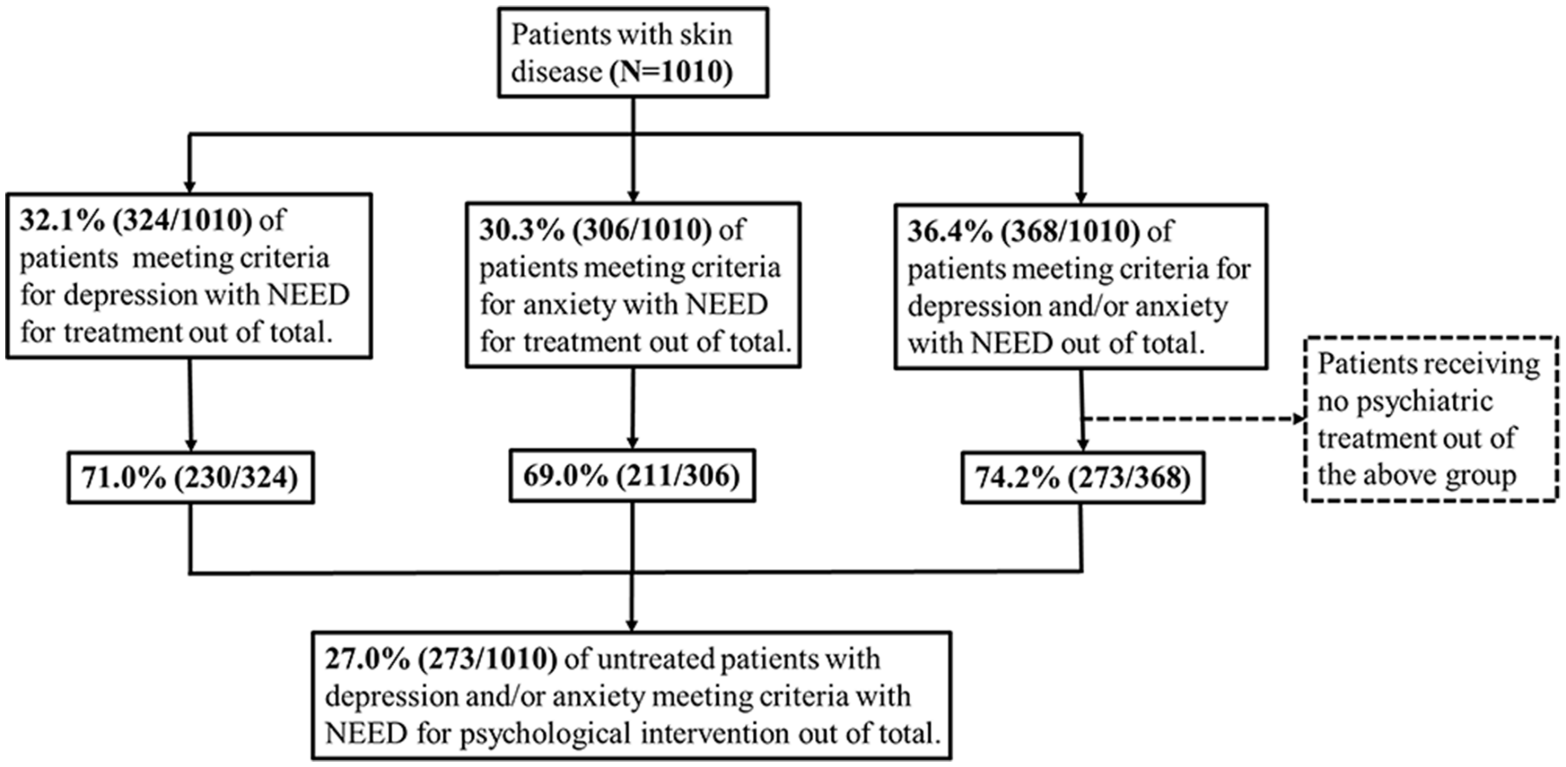
Flow chart of patients grouping based on their needs for mental health intervention for depression and/or anxiety and calculation of non-treatment rate (*N* = 1,010).

Overall, anxiety and depression were greatly associated (Spearman’s *β* = 0.756). The rates of depression and anxiety were 25.0% (252/1,010) and 23.2% (234/1,010), respectively. A total of 36.4% (368/1,010) of the patients met the criteria for needing mental health care; however, 74.2% (273/368) of them were not on any psychological care, indicating that 27.0% (273/1,010) of all the patients were not receiving any mental health care (Figure 1 and Supplementary Table 1).

### 3.2. Untreated psychological distress in patients needing mental health care

Among patients who met the criteria for needing mental health care, univariate analysis was performed between patients who received mental health care and those who were not treated for mental health problems. Patients who tend not to receive mental health care showed significantly lower income (OR: 0.80, 95%CI: 0.66–0.98), shorter duration of disease (OR: 0.61, 95%CI: 0.48–0.79), shorter delays in treatment (OR: 0.68, 95%CI: 0.54–0.87), and fewer symptoms on the face (OR: 0.57, 95%CI: 0.35–0.94). Patients with a higher level of adherence to dermatological treatment (OR: 1.65, 95%CI: 1.01–2.69) prefer seeking medical help in psychology.

Patients tend not to seek mental health care even presented more perceived stigmatization, poorer sleep quality (OR: 0.61, 95%CI: 0.38–0.99), and lower self-esteem (OR: 0.39, 95%CI: 0.24–0.64). Patients with suicide behaviors due to skin diseases (OR:1.31, 95%CI: 1.03–1.68) were found to seeking for medical help more often (Table 2). The multivariate regression analysis suggested that income (OR: 0.80, 95%CI: 0.65–0.99), duration of disease (OR: 0.68, 95%CI: 0.49–0.95), and suicide ideation (OR: 2.10, 95%CI: 1.14–3.87) were significant factors (Table 3).

**Table 2:** Factors of seeking for psychological interventions among patients with need for mental health care (*N* = 368).

Covariates	aOR^a^ (95% CI)
Socio-demographic feature	
Income	0.80 (0.66–0.98)*^b^
Clinical characteristics	
Duration of disease	0.61 (0.48–0.79)***
Facial symptoms	0.57 (0.35–0.94)*
Treatment delay	0.68 (0.54–0.87)**
Treatment adherence	1.65 (1.01–2.69)*
Medical costs	0.85 (0.68–1.06)
Psychological covariates due to skin disease	
Suicide ideation	1.02 (0.80–1.29)
Suicide behavior	1.31 (1.03–1.68)*
Stigmatization	0.49 (0.28–0.87)*
Poor sleep quality	0.61 (0.38–0.99)*
Lower self-esteem	0.39 (0.24–0.64)***

aOR, adjusted odds ratio.

^a^Logistic models were adjusted for age and sex; ^b^*P*-value represents the difference of covariates between psychologically treated and untreated patients.

* <0.05, ** <0.01, *** <0.001.

**Table 3:** Multivariate regression analysis for predictors of mental health care seeking (*N* = 368).

	β	aOR^a^ (95%CI)
Income	−0.227	0.80 (0.65–0.99)*
Duration of disease	−0.386	0.68 (0.49–0.95)*
Suicide ideation	0.742	2.10 (1.14–3.87)*
Constant	1.956	7.07

^a^Logistic models were adjusted for age and sex.

* <0.05.

### 3.3. Reasons for non-treatment of psychological distress in patients with skin diseases

**Figure 2:**
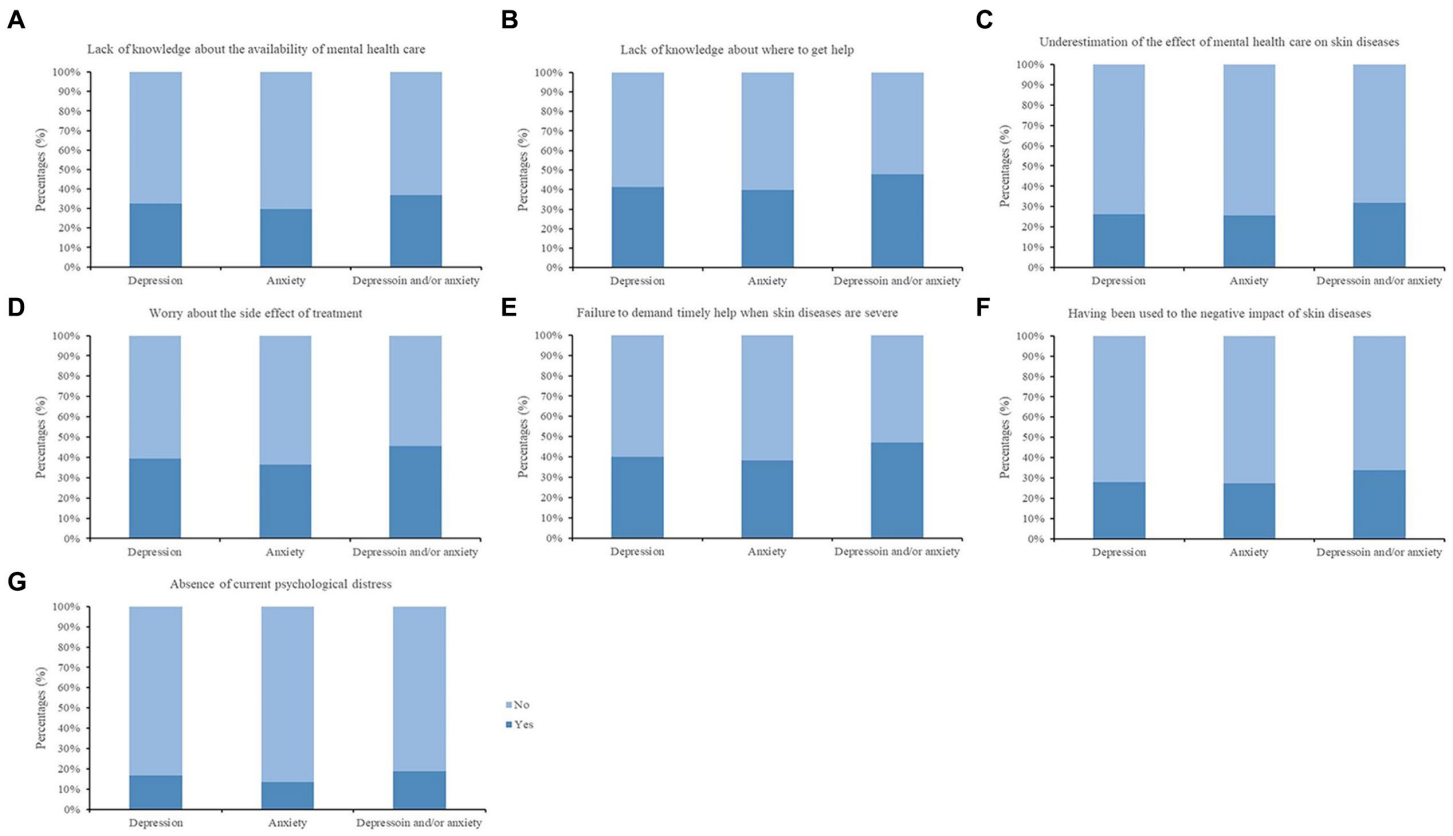
Reasons for non-treatment in patients receiving no mental health care (*N*=895). Depression: patients with a 9-Item Patient Health Questionnaire (PHQ-9) score ≥10 or patients with a lower score but receiving mental health care. Anxiety: patients with a Generalized Anxiety Disorder-7 (GAD-7) score ≥8 or patients with lower scores but receiving mental health care. Depression and/or anxiety: patient with a PHQ-9 ≥10 and/or a GAD-7 score ≥8, or with a lower score for either scale but receiving mental health care. Reasons for non-treatment were followed. **(A)** Lack of knowledge about the availability of mental health care; **(B)** Lack of knowledge about where to get help; **(C)** Underestimation of the effect of mental health care on skin diseases; **(D)** Worries about the side effects of treatment; **(E)** Failure to seek help timely when their skin diseases became severe; **(F)** Having been used to the negative impact of skin diseases; **(G)** Absence of current psychological distress.

Among patients who did not receive mental health services, the leading reason for non-treatment for their psychological distress was underestimation of its effect on their skin diseases, while the least selected reason was absence of current psychological distress (Figure 2 and Supplementary Table 2). The association between the reasons for psychological non-treatment and the need for psychological intervention was demonstrated in the patients who received no mental health care; the need for mental health care was positively associated with the lack of knowledge about the availability of mental health services (OR: 1.46, 95%CI: 1.06–2.00), lack of knowledge about where to get help (OR: 2.73, 95%CI: 1.99–3.74), worries about the side effects of treatment (OR: 2.21, 95%CI: 1.56–3.14), and failure to seek help timely when the skin disease became severe (OR: 2.21, 95%CI: 1.48–3.30), and was negatively associated with the absence of current psychological distress (OR: 0.52, 95%CI: 0.30–0.89) (Table 4).

**Table 4:** Association between the need for psychological intervention and the reasons for non-treatment in patients receiving no mental health care (*N* = 895).

	Total (*N* = 895)	Subgroup A (*N* = 499)	Subgroup B (*N* = 396)
Lack of knowledge about the availability of mental health care (*N* = 238)	1.46 (1.06–2.00)*	1.04 (0.67–1.62)	2.22 (1.39–3.55)**
Lack of knowledge about where to get help (*N* = 226)	2.73 (1.99–3.74)***	2.66 (1.71–4.13)***	2.88 (1.81–4.59)***
Underestimation of the effect of mental health care on skin diseases (*N* = 343)	1.12 (0.84–1.51)	1.08 (0.71–1.64)	1.15 (0.75–1.75)
Worries about the side effects of treatment (*N* = 165)	2.21 (1.56–3.14)***	2.27 (1.43–3.61)**	2.19 (1.26–3.81)**
Failure to seek help timely when their skin diseases became severe (*N* = 115)	2.21 (1.48–3.30)***	1.70 (0.98–2.96)	3.08 (1.69–5.62)***
Having been used to the negative impact of skin diseases (*N* = 252)	1.23 (0.90–1.68)	1.00 (0.63–1.57)	1.48 (0.95–2.31)
Absence of current psychological distress (*N* = 96)	0.52 (0.30–0.89)*	0.79 (0.40–1.55)	0.29 (0.12–0.70)**

Subgroup A: patients who were adherent to treatment, or there was no treatment prescribed (*N* = 499). Subgroup B: patients who were not adherent to treatment (*n* = 396).

Data were adjusted odds ratio (95%CI). Logistic models were adjusted for age and sex. Association between the need for psychological intervention and the reasons for non-treatment were analyzed in all patients (*N* = 895). Subgroup analysis was also conducted for patients who were adherent to dermatological treatment/with no treatment prescribed (*N* = 499) and patients not adherent to dermatological treatment (*N* = 396).

* <0.05, ** <0.01, *** <0.001.

As we found that the adherence to dermatological treatment was associated with patients’ mental health care seeking behaviors (Table 2), we performed the subgroup analysis on the need for mental health care and the reasons for non-treatment. The result showed that, among patients adherent to their dermatological treatment, the lack of knowledge about where to get help (OR: 2.66, 95%CI: 1.71–4.13) and worries about the side effects of treatment (OR: 2.27, 95%CI: 1.43–3.61) were significant factors (Table 4).

## 4. Discussion

Compared to studies focusing on the mental health status only in patients with skin diseases, the present study also identified some of the reasons for these patients’ non-treatment and neglected needs for mental health care, as well as explored the field of psychodermatology from perspectives of patient perception and medical consensus. We found that, among patients with skin diseases, a large percentage of them who needed mental health care were not on psychological treatment, and the patients’ higher need for treatment was associated with a lower income, longer duration of disease, and suicide ideation. We also found that a lack of knowledge about where to get help and worries about the side effects of treatment were significantly associated with the need for psychological intervention.

### 4.1. High prevalence of psychological nontreatment in patients with skin diseases

In the present study, the rates of depression and anxiety were 25.0 and 23.2%, respectively, and 36.4% of the patients with skin diseases who needed mental health care were not on such treatment, accounting for 27.0% of all the participants. In line with our findings, a study in Brazil found that, among patients with psoriasis, 35.2 and 35.6% had depression and anxiety, respectively (Pollo et al., 2021). A study in England reported that most patients (82%) receiving no psychological intervention had poorer emotional and psychological wellbeing (Edward, 2020). A study in America in 2016 also suggested a high percentage (38.8%) of patients with skin diseases who needed mental health intervention as well as an alarming percentage (14.4%) of patients who received no mental health care (Achtman et al., 2016). All the above findings reflect that the need for mental health care in patients with skin diseases has been largely neglected, indicating the importance of timely treatment.

### 4.2. Unmet need for psychological intervention

Mental health services are severely underutilized in China; however, factors affecting mental health care seeking behaviors in patients with skin diseases have not been fully identified. In the present study, we found that patients having a lower income were more likely to receive psychological intervention. Some studies also found that, compared with patients with a higher income, who received more psychological intervention (Lu et al., 2021), those having a lower income were more impacted by their skin diseases (Cheng et al., 2021; Zhang et al., 2022). Therefore, the patients with lower income may suffer from more psychological distress with the aggravation of skin diseases, which leads to higher seeking for mental health care.

Chronic skin diseases adversely affect all aspects of life of patients. With longer duration of their skin diseases as well as treatment delay, patients with psychological distress are more likely to have no psychological help. Patients can perceive a higher level of stigma about skin diseases without seeking mental health care. A study suggested that higher skin lesions, e.g., lesions on the face, might lead to more perceived stigma of the skin diseases (Germain et al., 2021). It was also noted that patients with a higher level of stigma of skin conditions preferred unnecessarily frequent visits to dermatologists rather than seeing a psychiatrist (Schut et al., 2022). Thus, dermatologists may need to consider psychological factors when reviewing their patients.

It’s too late that patients were given more mental health care when presented with suicidal behaviors. A study found that 23.5% of the subjects who had received psychological intervention engaged in suicidal behaviors. A study conducted in European countries showed that up to 12.7% of all outpatients with skin diseases had suicide ideation (Dalgard et al., 2015), and a study in China found that an even more alarming percentage (34%) of patients with psoriasis had suicide ideation (Chen et al., 2019). The above findings suggested that patients with skin diseases may suffer from chronic psychological distress in the absence of life-threatening conditions.

### 4.3. Underutilization of mental health care by patients with skin diseases

According to the present study, the lack of knowledge about the availability of mental health services and the failure to seek timely treatment in patients with skin diseases have reflected the lack of public knowledge about psychodermatology. The primary health care service programs in China suggest that the awareness rate of mental health management is still very low, at 11–38% (Magid and Reichenberg, 2020), and that the awareness rate is even lower among people with a lower level of education and those who have never had contact with patients with mental disorders (Zhou et al., 2019). In the present study, nearly one third of patients who did not receive mental health care indicated that they had been used to the negative psychological impact of their skin diseases; another study recommended that patients with skin diseases should also receive mental health care (Hao et al., 2019), as patients who have not received timely mental health care are more prone to the sense of helplessness and a lower perceived ability to cope with the disease (Evers et al., 2005). As the awareness for mental health care needs to be improved for patients with skin diseases and mental health issues, educational columns on knowledge about such diseases should be established, and public education also needs to focus on reducing public prejudice toward mental health intervention.

For all patients with skin diseases, regardless of whether they are on regular treatment, the more they worry about the side effects of mental health treatment and they are unclear about where to seek psychological help, the more they need psychological intervention. Patients overwhelmed by worries about the side effects of psychotropic medication are more likely to neglect the consequences of untreated mental health issues, thinking that self-management of their issues can substitute for psychological intervention. In the present study, only 10.7% (96/895) of the participants who did not receive mental health care indicated that they had no psychological distress for the time being. Furthermore, the small scope of public education by relevant medical organizations has also limited the availability of medical resource, and mental health care professionals are often part-time or casual employees in the community, which further affects the consistency of treatment (You and Lian, 2021). A study has found that patients who have received psychological intervention are still not active in seeking treatment (Xiao et al., 2017). All the above factors not only limited the quality of treatment at community level, but also reduced the public trust in mental health institutions, thereby reducing public utilization of mental health services.

Distrust in medical institutions and perceived stigma toward psychiatric treatment are prevalent (Qi et al., 2021; Thornicroft et al., 2022), making patients with skin diseases even more reluctant to seek psychological help. Our findings indicated that a large number of patients believed that mental health care might not be effective on skin diseases. Furthermore, the current routes of medical treatment seeking and available medical resources have limited the effective popularization and implementation of mental health services; thus, the improvement of professional awareness, the professionalism of health care workers and the quality of institutions need further exploration and discussion, in order to ultimately improve the quality of treatment.

Several limitations should be noted. First, this retrospective study might have led to recall bias. Second, depression and anxiety were assessed by the PHQ-9 and GAD-7 self-rating scales rather than via clinical interview, making the results more susceptible to subjective influence. However, both of the screening scales had been calculated to be valid and reliable (Wang et al., 2014; Tong et al., 2016; Tian et al., 2019; Huang et al., 2020). Third, data were collected from patients with common skin disease and so may not be generalizable to all skin conditions. In addition, more information on the duration of dermatological treatment should be described. Although patients whether being adherence to the dermatological treatment or not were divided into two subgroups and different significance was found. Future studies need to be strengthened on specific skin diseases and severity of skin disease.

## 5. Conclusion

Through the investigation of the need for psychological intervention among patients with skin diseases as well as the reasons why this need is unmet, we found that patients with skin diseases have great needs for psychological intervention, but they are less likely to seek mental health services due to the lack of knowledge about their diseases and necessary treatments. In the future, extensive public education and special columns are needed to improve public awareness of diseases and treatment. Meanwhile, attention to and professional recognition of psychodermatology by medical institutions, training of interdisciplinary professionals, and the quality of treatment by mental health institutions should also be enhanced, in order to improve the physical and mental health of patients with skin diseases.

## Data availability statement

The original contributions presented in the study are included in the article/Supplementary material, further inquiries can be directed to the corresponding author.

## Ethics statement

The studies involving human participants were reviewed and approved by the Ethics Committee of Xiangya Hospital, Central South University (Changsha, China). The patients/participants provided their written informed consent to participate in this study.

## Author contributions

S-LZ, JS, and MW contributed to conception and design of the study and funded the study. T-RT and HL organized the database and performed the statistical analyses. S-CY and C-CZ conducted secondary checks of data and contributed to manuscript amendments. T-RT wrote the first draft of the manuscript. MW corrected the manuscript. All authors contributed to manuscript revision, read, and approved the final version.

## Funding

This work was supported by a grant from Health Commission of Hunan Province in China (grant no. B202314010044).

## Conflict of interest

The authors declare that the research was conducted in the absence of any commercial or financial relationships that could be construed as a potential conflict of interest.

## Publisher’s note

All claims expressed in this article are solely those of the authors and do not necessarily represent those of their affiliated organizations, or those of the publisher, the editors and the reviewers. Any product that may be evaluated in this article, or claim that may be made by its manufacturer, is not guaranteed or endorsed by the publisher.
