# Corrigendum: Untreated depression and anxiety in patients with common skin diseases: a cross-sectional study in China

**DOI:** 10.3389/fpsyg.2023.1340646

**Published:** 2023-11-30

**Authors:** Tao-Ran Tang, Mi Wang, Hong Li, Song-Chun Yang, Cheng-Cheng Zhang, Wen-Rui Lin, Xin-Chen Ke, Han-Yi Zhang, Juan Su, Shi-Lin Zhu

**Affiliations:** ^1^School of Nursing, Hunan University of Chinese Medicine, Hunan, Changsha, China; ^2^Department of Dermatology, Xiangya Hospital, Central South University, Hunan, Changsha, China; ^3^Department of Social Medicine and Health Management, School of Public Health, Central South University, Hunan, Changsha, China; ^4^The Second Affiliated Hospital, Hunan University of Chinese Medicine, Hunan, Changsha, China

**Keywords:** depression, anxiety, skin disease, mental health service, web-based, survey

In the published article, there was an error in the Funding statement. The grant number was stated as “B202314017099” but should be “B202314010044”. The correct Funding statement appears below.

