# Corrigendum: Untreated depression and anxiety in patients with common skin diseases: a cross-sectional study in China

**DOI:** 10.3389/fpsyg.2024.1368480

**Published:** 2024-02-07

**Authors:** Tao-Ran Tang, Mi Wang, Hong Li, Song-Chun Yang, Cheng-Cheng Zhang, Wen-Rui Lin, Xin-Chen Ke, Han-Yi Zhang, Juan Su, Shi-Lin Zhu

**Affiliations:** ^1^School of Nursing, Hunan University of Chinese Medicine, Hunan, Changsha, China; ^2^Department of Nursing, The Second Affiliated Hospital of Hunan University of Chinese Medicine, Hunan, Changsha, China; ^3^Department of Dermatology, Xiangya Hospital, Central South University, Hunan, Changsha, China; ^4^Department of Social Medicine and Health Management, School of Public Health, Central South University, Hunan, Changsha, China

**Keywords:** depression, anxiety, skin disease, mental health service, web-based, survey

In the published article, there were errors in affiliations 2, 3, and 4. For affiliation 2, instead of “Department of Dermatology, Xiangya Hospital, Central South University, Hunan, Changsha, China,” it should be “Department of Nursing, The Second Affiliated Hospital of Hunan University of Chinese Medicine, Hunan, Changsha, China.” For affiliation 3, instead of “Department of Social Medicine and Health Management, School of Public Health, Central South University, Hunan, Changsha, China,” it should be “Department of Dermatology, Xiangya Hospital, Central South University, Hunan, Changsha, China.” For affiliation 4, instead of “The Second Affiliated Hospital, Hunan University of Chinese Medicine, Hunan, Changsha, China,” it should be “Department of Social Medicine and Health Management, School of Public Health, Central South University, Hunan, Changsha, China.”

Overall, instead of “1 School of Nursing, Hunan University of Chinese Medicine, Hunan, Changsha, China, 2 Department of Dermatology, Xiangya Hospital, Central South University, Hunan, Changsha, China, 3 Department of Social Medicine and Health Management, School of Public Health, Central South University, Hunan, Changsha, China, and 4 The Second Affiliated Hospital, Hunan University of Chinese Medicine, Hunan, Changsha, China,” it should be “1 School of Nursing, Hunan University of Chinese Medicine, Hunan, Changsha, China, 2 Department of Nursing, The Second Affiliated Hospital of Hunan University of Chinese Medicine, Hunan, Changsha, China, 3 Department of Dermatology, Xiangya Hospital, Central South University, Hunan, Changsha, China, and 4 Department of Social Medicine and Health Management, School of Public Health, Central South University, Hunan, Changsha, China.”

Additionally, there were errors regarding the affiliations noted for author Tao-Ran Tang, Mi Wang, Song-Chun Yang, Cheng-Cheng Zhang, Wen-Rui Lin, Xin-Chen Ke, Han-Yi Zhang, Juan Su, Shi-Lin Zhu. For the affiliations noted for author Tao-Ran Tang. As well as having affiliation 1, “School of Nursing, Hunan University of Chinese Medicine, Hunan, Changsha, China”, they should also have the new affiliation 2, “Department of Nursing, The Second Affiliated Hospital of Hunan University of Chinese Medicine, Hunan, Changsha, China”. For the affiliation noted for author Mi Wang, Song-Chun Yang, Wen-Rui Lin, Xin-Chen Ke, Han-Yi Zhang, Juan Su. Instead of having affiliation 2, “Department of Dermatology, Xiangya Hospital, Central South University, Hunan, Changsha, China”, they should have the new affiliation 3, “Department of Dermatology, Xiangya Hospital, Central South University, Hunan, Changsha, China”. For the affiliation noted for author Cheng-Cheng Zhang. Instead of having affiliation 3, “Department of Social Medicine and Health Management, School of Public Health, Central South University, Hunan, Changsha, China”, they should have the new affiliation 4, “Department of Social Medicine and Health Management, School of Public Health, Central South University, Hunan, Changsha, China”. For the affiliations noted for author Shi-Lin Zhu. Instead of having affiliation 4, “The Second Affiliated Hospital, Hunan University of Chinese Medicine, Hunan, Changsha, China”, they should have the new affiliation 2, “Department of Nursing, The Second Affiliated Hospital of Hunan University of Chinese Medicine, Hunan, Changsha, China”.

Overall, instead of “Tao-Ran Tang^1^, Mi Wang^2^, Hong Li^1^, Song-Chun Yang^2^, Cheng-Cheng Zhang^3^, Wen-Rui Lin^2^, Xin-Chen Ke^2^, Han-Yi Zhang^2^, Juan Su^2^, Shi-Lin Zhu^4*^”, they should be “Tao-Ran Tang^1, 2^, Mi Wang^3^, Hong Li^1^, Song-Chun Yang^3^, Cheng-Cheng Zhang^4^, Wen-Rui Lin^3^, Xin-Chen Ke^3^, Han-Yi Zhang^3^, Juan Su^3^, Shi-Lin Zhu^2*^”.

The authors apologize for these errors and state that they do not change the scientific conclusions of the article in any way. The original article has been updated.

