# Supplementary material for: Untreated depression and anxiety in patients with common skin diseases: a cross-sectional study in China
Source: Front Psychol. 2023 May 16;14:1150998. doi: 10.3389/fpsyg.2023.1150998 (PMC10227509; doi:10.3389/fpsyg.2023.1150998)
Supplement: Supplementary file 1 [file Data_Sheet_1.docx]

Supplementary Material

Untreated Depression and Anxiety in Patients with Common Skin Diseases: A Cross-sectional Study in China

**Tao-Ran Tang, Mi Wang, Hong Li, Song-Chun Yang, Cheng-Cheng Zhang, Wen-Rui Lin, Xin-Chen Ke, Han-Yi Zhang, Juan Su and Shi-Lin Zhu***

**Correspondence:** Shi-Lin Zhu: zhushilin@hnucm.edu.cn

# Supplementary Tables

**
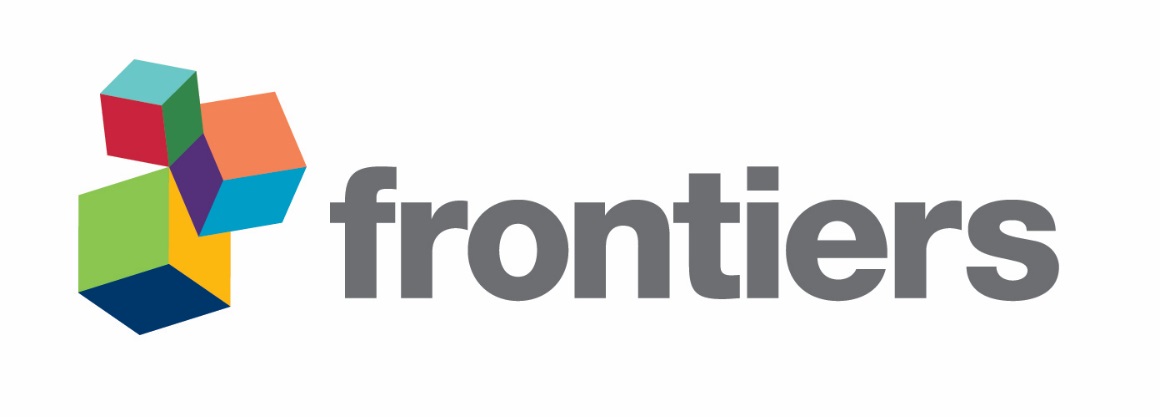
**

**Supplementary Table 1.** Percentage of patients with depression and/or anxiety who needed psychological intervention and the non-treatment rate (N=1,010).

| **Correlation** |  |
| --- | --- |
| Spearman 's β for depression and anxiety (*p* <0.001) | r=0.756*** |
| **Depression** |  |
| Percentage of patients who needed psychological intervention | 32.1% (324/1,010) |
| Percentage of patients receiving no psychological intervention in this patient group | 71.0% (230/324) |
| **Anxiety** |  |
| Percentage of patients who needed psychological intervention | 30.3% (306/1,010) |
| Percentage of patients receiving no psychological intervention in this patient group | 69.0% (211/306) |
| **Depression and/or anxiety** |  |
| Percentage of patients who needed psychological intervention | 36.4% (368/1,010) |
| Percentage of patients receiving no psychological intervention in this patient group | 74.2% (273/368) |
| Percentage of untreated patients with depression and/or anxiety who needed psychological intervention in the whole sample | 27.0% (273/1,010) |

*** <0.001

**Supplementary Table 2.** Percentage of patients with depression and/or anxiety who needed psychological intervention and the non-treatment rate (N=1,010).

|  | Total | Depression | Anxiety | Depression and/or anxiety |
| --- | --- | --- | --- | --- |
| Lack of knowledge about the availability of mental health care, N (%) | 238 | 127 (42.5%) | 122 (40.8%) | 139 (46.5%) |
| Lack of knowledge about where to get help, N (%) | 226 | 125 (47.3%) | 122 (46.2%) | 140 (53.0%) |
| Underestimation of the effect of mental health care on skin diseases, N (%) | 343 | 119 (31.3%) | 118 (31.1%) | 139 (36.6%) |
| Worries about the side effects of treatment, N (%) | 165 | 86 (45.0%) | 81 (42.4%) | 96 (50.3%) |
| Failure to seek help timely when their skin diseases became severe, N (%) | 115 | 62 (45.9%) | 60 (44.4%) | 70 (51.9%) |
| Having been used to the negative impact of skin diseases, N (%) | 252 | 86 (31.6%) | 85 (31.3%) | 101 (37.1%) |
| Absence of current psychological distress, N (%) | 96 | 17 (17.3%) | 14 (14.3%) | 19 (19.4%) |

PHQ-9: 9-Item Patient Health Questionnaire. GAD-7: Generalized Anxiety Disorder-7.

Depression: patients with a PHQ-9 score ≥10 or patients with a lower score but receiving mental health care. Anxiety: patients with a GAD-7 score ≥8 or patients with lower scores but receiving mental health care. Depression and/or anxiety: patient with a PHQ-9 ≥10 and/or a GAD-7 score ≥8, or with a lower score for either scale but receiving mental health care.
